# Technology of Combined Identification of Macrophages and Collagen Fibers in Liver Samples

**DOI:** 10.17691/stm2024.16.3.03

**Published:** 2024-06-28

**Authors:** I.A. Nikitina, V.A. Razenkova, E.A. Fedorova, O.V. Kirik, D.E. Korzhevskii

**Affiliations:** Junior Researcher, Laboratory of Experimental Histology and Confocal Microscopy, Department of General and Special Morphology; The Institute of Experimental Medicine, 12 Akademika Pavlova St., Saint Petersburg, 197376, Russia; Junior Researcher, Laboratory of Functional Morphology of Central and Peripheral Nervous System, Department of General and Special Morphology; The Institute of Experimental Medicine, 12 Akademika Pavlova St., Saint Petersburg, 197376, Russia; PhD, Researcher, Laboratory of Functional Morphology of Central and Peripheral Nervous System, Department of General and Special Morphology; The Institute of Experimental Medicine, 12 Akademika Pavlova St., Saint Petersburg, 197376, Russia; PhD, Senior Researcher, Laboratory of Functional Morphology of Central and Peripheral Nervous System, Department of General and Special Morphology; The Institute of Experimental Medicine, 12 Akademika Pavlova St., Saint Petersburg, 197376, Russia; MD, DSc, Professor of the Russian Academy of Sciences, Head of the Laboratory of Functional Morphology of Central and Peripheral Nervous System; Head of the Department of General and Special Morphology; The Institute of Experimental Medicine, 12 Akademika Pavlova St., Saint Petersburg, 197376, Russia

**Keywords:** liver, macrophages, Kupffer cells, Iba-1

## Abstract

The importance of identifying pathological changes in the liver both in fundamental researches and in diagnostic practice dictates the necessity to have a convenient method of assessing functional condition of resident macrophages and connective tissue fibers.

**The aim of the study** is to assess the technology of combined histo-immunohistochemical detection of collagen fibers of connective tissue and resident liver macrophages using aniline blue histological stain and available antibodies to the microglial marker, the Iba-1 protein.

**Materials and Methods:**

Liver samples from adult rats (n=6) have been used in the study. The connective tissue was stained with a 2% aqueous solution of aniline blue (Unisource Chemicals Ltd., India). Monoclonal rabbit antibodies to Iba-1 (Clone JM36-62; ET1705-78; HuaBio, China) were used to detect resident liver macrophages, zinc-ethanol-formaldehyde was employed as a fixative.

**Results:**

The combined staining method allowed us to detect numerous Iba-1-immunopositive structures corresponding morphologically to Kupffer cells and connective tissue macrophages, background staining was not observed. Staining with aniline blue in the liver samples was selective, uniform, and clear, and allowed for differentiation of the connective tissue in all examined samples. Exclusion of the heat-induced epitope retrieval stage caused no negative effect on identification of macrophages, reduced the probability of non-specific staining of the collagen fibers with aniline blue, and ensured general preservation of tinctorial properties of the liver tissue.

**Conclusion:**

The presented protocol of combined histo-immunohistochemical identification of Kupffer cells and connective tissue fibers, applied on the rat liver samples, makes it possible to perform effectively the morphometric analysis and may find its application in pathohistological, clinical, and preclinical investigations.

## Introduction

Kupffer cells are resident liver macrophages, which are in close contact with sinusoidal capillaries of the liver and play an important role in the mononuclear phagocyte system of the body. These cells perform various functions: phagocytosis of cell debris and toxins entering the liver via the portal vein; involvement in lipid metabolism (including cholesterol) and proteins; provision of immunosurveillance; maintenance and regulation of immune tolerance of the body [1]. Besides, one of the important functions of the Kupffer cells is their interaction with fibroblasts and myofibroblasts, the cells responsible for synthesis and secretion of collagen precursors.

Recent investigations have shown that proimflammatory activation of macrophages in various tissues and organs induce the release of interleukins IL-4 and IL-13 and profibrotic factors (TGF-β1, FGF-2, PDGF) stimulating epithelial-mesenchymal transformation and deposition of extracellular matrix production. This process results in remodeling of the extracellular matrix of the connective tissue and pathologic angiogenesis, which, in their turn, force persistent hyperactivation of fibroblasts and myofibroblasts [2]. Thus, one of the functions of macrophages in organs and tissues (Kupffer cells in the liver, in particular) is profibrogenic regulation.

The development of new universal approaches, which combine the advantages of classic histological methods of staining and immunohistochemical reactions, is of great importance for histological practice. Simultaneous exploration of the functional condition of the Kupffer cells and connective tissue provides the possibility to study the mechanisms of pathological changes developing in the liver. The most commonly applied methods for exploring the connective tissue on preparations are histological methods of Van Gieson’s staining and Mallory and Masson trichrome staining with aniline blue [3–7]. These methods can be used to stain the liver tissue both in normal and pathological conditions. However, the Kupffer cells cannot be detected by the classical histological staining techniques. It has been previously shown [8] that it is convenient to use immunohistological reaction to Iba-1 microglial protein. Therefore, to identify concurrently the connective tissue and Kupffer cells it is appropriate to combine histological staining of collagen fibers with aniline blue and immunohistochemical identification of the Kupffer cells with reaction to the Iba-1 protein. It is worth mentioning that possible changes in the tissue properties due to stain absorption during successive material treatment for the two variants of the investigation make the result of this approach unobvious.

In this connection, **the aim of our study** was to assess the possibility of using a combined approach to concurrent detection of Kupffer cells and a fibrous component of the connective tissue on the liver samples.

## Materials and Methods

The study was performed on the liver samples of adult (4–6 months) Wistar (n=3) and SHR (n=3) rats. The rats were delivered from the nurseries for laboratory animals “Rappolovo” (Leningrad region, Russia) and “Pushchino” (Moscow region, Russia), were housed in vivarium at room temperature, under standard conditions, with a free access to food and water. Housing and scarification of animals complied with the ethical principles of the European Convention for the Protection of Vertebrate Animals used for Experimental and Other Scientific Purposes (Strasbourg, 2006) and Order No.199n “On the Approval of the Rules of Good Laboratory Practice” (Russia, 2016). During the investigations, all international principles of using animals were observed. The study was approved by the local Ethical Committee of the Institute of Experimental Medicine (Saint Petersburg, Russia).

The left liver lobe was used for the investigation. The liver samples were fixed in zink-ethanol-formaldehyde [9] for 18–24 h at room temperature. The fixed material was embedded into paraffin according to the standard protocol and blocks containing one liver lobe were fabricated. The paraffin blocks were cut into 5 μm sections using rotation Microm HM 325 microtome (Thermo Fisher Scientific, USA), which were mounted on the HistoBond®+M adhesive microscope slides (Marienfeld, Germany). Further, standard procedures of dewaxing and dehydration were conducted.

Monoclonal rabbit antibodies to Iba-1 (Clone JM36-62; ET1705-78; HuaBio, China) were used to detect resident liver macrophages. UltraVision Quanto Detection System HRP DAB (Thermo Fisher Scientific, USA) was employed as a secondary reagent for primary rabbit antibodies. The slices were stained with a 2% aqueous solution of aniline blue (Unisource Chemicals Pvt. Ltd., India) being a component of the Mallori and Masson trichrome stain acidified with glacial acetic acid. To stain the slices, first, a mordant (phosphomolybdic acid) was applied followed by a freshly prepared solution of aniline blue.

After dehydration in isopropanol and bleaching in orthoxylol, the obtained preparations were placed into the permanent Cytoseal 60 mounting medium (Richard-Allan Scientific, USA) and analyzed using a light Axio Scope.A1 microscope (Carl Zeiss, Germany). Photographs of the histological preparations were taken using Zeiss Axiocam 105 color camera (A-Plan 20×/0.45; 40×/0.65 objectives) and ZEN 3 program (Carl Zeiss, Germany).

The obtained images were morphometrically analyzed using ImageJ2 program with FIJI distribution [10]. To assess quantitatively interlobular connective tissue and the distribution density of Iba-1 positive elements, the images were presegmented by 4 colors (red, yellow, blue, white) using IJ-Plugins Toolkit [11] and k-means algorithm. As the result of segmentation, binarized images corresponding to the examined structures have been obtained. The total area of the Iba-1 immunostained structures and collagen fibers was evaluated with the help of the standard ImageJ2 functions such as color histogram, analysis of particles, and measurement. A morphometric grid with a specified point density (11×11), which was applied to the image separately using the GIMP graphics editor, was also employed [12].

To quantitatively evaluate the density of Iba-1 positive element distribution associated with interlobular connective tissue, regions of interest were preselected with the help of the standard ImageJ2 function “region of interest”. Next, by means of IJ-Plugins k-means clustering [13], images were segmented by 3 colors (RGB). The total area of the Iba-1 immunostained structures was estimated using the above mentioned ImageJ2 functions for morphometric analysis. The measured area of the objects in the image was expressed in square micrometers and in percentages.

## Results

In the course of the preliminary research, we have assessed the possibility of staining the collagen fibers with aniline blue after setting up the immunohistochemical reaction to the Iba-1 protein considering the previously developed protocol [8] which requires heat-induced epitope retrieval (HIER). The test results have shown that after HIER, the collagen fibers were detected non-selectively, and in this connection, the protocol has been modified. Testing different modes of primary reagent incubation resulted in exclusion of the HIER stage, reduction of the period of primary reagent incubation (from 3 to 1 day), and elevation of the incubation temperature (from 27.5 to 35°C). This version of the protocol allowed us to achieve optimal results of immunohistochemical reaction.

**Figure 1.**
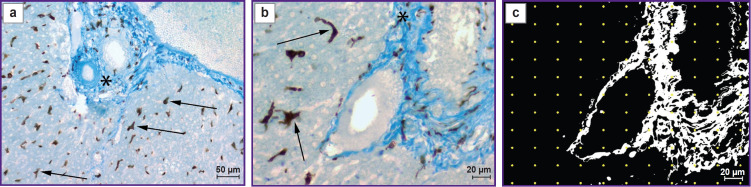
The result of staining the connective tissue with aniline blue with identification of Kupffer cells using immunohistochemical reaction to the Iba-1 protein and the quantitative analysis of collagen fibers by means of the morphometric grid: (a), (b) Iba-1-immunopositive cells and collagen fibers with aniline blue staining in Wistar rat liver samples; (c) binary transformation of the (b) image using color segmentation for differentiation of the collagen fibers and demonstration of the point version of the morphometric grid. Arrows show Iba-1 immunopositive cells, asterisks show collagen fibers. Objectives: A-plan 20×/0.45 (a); A-plan 40×/0.65 (b), (c)

As the result of the reaction to the Iba-1 protein, multiple Iba-1 immunopositive structures had morphological features that matched to the Kupffer cells (Figure 1 (a), (b)) have been identified in all examined samples. No background staining was observed. The detected cells were morphologically similar, clear and mostly uniform staining of cytoplasma was noted. In some cases, the site of nucleus location was seen. Presence of projections, well visualized due to intensive staining, was characteristic for the majority of Kupffer cells. In all examined samples, these projections contacted with vascular endothelial cells, hepatocytes and other connective cells in the region of periportal laminar boundary layer. Exclusion of the HIER caused no negative effect on the detection of macrophages, reduced the possibility of nonspecific staining and improved the preservation of the liver tissue samples in the process of section treatment. At the same time, the elevation of incubation temperature allowed us to decrease the time of holding the sections in the solution of primary antibodies. Visually, the immunohistochemical reaction to the Iba-1 protein in all the examined samples was highly intensive and did not prevent selective staining of the collagen with aniline blue.

Staining with aniline blue in all liver samples of Wistar and SHR rats was selective, uniform, and clear and allowed for differentiation of the connective tissue in all sections. Treatment of the slices with phosphomolybdic acid and staining with aniline blue after immunohistochemical reaction to the Iba-1 did not influence negatively the preservation of the product of the DAB chromogen reaction. Reduction in the staining intensity of the immunohistochemical reaction product or washing it out from the sections has not been noted.

The combined staining method allowed hereafter for estimating morphometrically the area of immunopositive structures and the area occupied by the collagen fibers in the ocular view (Figure 1 (c)).

The image, presented in Figure 1 (b) was used as an example for quantitative analysis. Thus, the area, occupied by the collagen fibers, was calculated using a morphometric grid. The estimated area was 11,617.24 μm^2^ (20.66% of the total area of the image). The total area of the Iba-1 immunostained structures was calculated on the basis of binarized image using a color histogram and was equal to 2330.08 μm^2^ (4.15% of the total area of the image).

**Figure 2.**
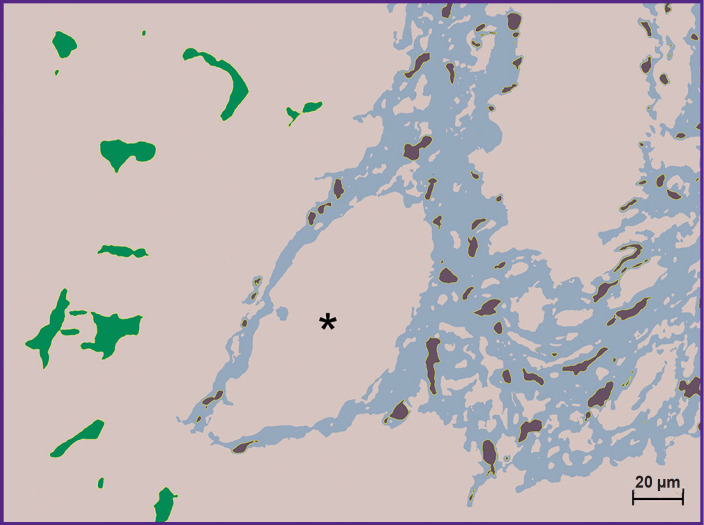
The result of image segmentation based on the segregation of the color values by the clusters using k-means clustering plugin for ImageJ2 Green color shows Kupffer cells; brown designates macrophages associated with the fibers of the interlobular connective tissue; the central vein is shown by the asterisk

The macrophages associated with interlobular connective tissue were automatically segregated using ImageJ2 k-means clustering plugin (Figure 2). This plugin enabled us selectively estimate the total area of macrophages of interlobular connective tissue and the Kupffer cells and also to determine the number of cells and cell fragments based on the color image. The total area of macrophages of the interlobular connective tissue was 1013.61 μm^2^ (1.80%), and the number of detected cell fragments — 76. The total area of the Kupffer cells was 1316.47 μm^2^ (2.34%), the total number of the cells and their fragments — 16.

## Discussion

Fibrosis and activation of the immune system cells (the resident liver macrophages, Kupffer cells, in particular) accompany the majority of chronic liver diseases. In the diagnostic practice, it is often possible to establish the form of fibrosis only by the results of histological investigations [3, 14]. This investigation is necessary for the development of the biological models of fibrosis [15], at the preclinical stage of developing new medicinal preparations [16], and during clinical trials [3, 4]. In the present investigation, we have optimized the stages of setting up the immunohistochemical reaction in the previously proposed protocol for Kupffer cell detection using antibodies to microglial Iba-1 marker [8], which allowed for the application of aniline blue for staining histological sections. To enhance the specificity of collagen fiber staining after immunohistochemical investigation and preservation of tinctorial properties of the tissue, the possibility to remove the HIER was considered.

The HIER procedure is used rather frequently in immunohistochemical investigations, since it enhances the sensitivity of the method [17], however, direct tissue heating may change the tinctorial properties of the examined tissue, which distorts the results of the following histological staining. Thus, it has been shown that denaturation of polypeptide chains and breaking of the bonds between them occur during heating process. Collagen denaturation is a multistage process accompanied by the impairment of the specific configuration of the glycine, proline, and alanine molecules [18, 19].

Exclusion of antigen retrieval stage in the presented technique of immunohisochemical staining allowed us to avoid denaturation of the collagen on the sections, which provide the possibility to study tissue macrophages and detect connective tissue fibers within the limits of one section. It gives the researcher the tools for exploring their mutual arrangement and a more precise assessment of the functional state of the organ.

When immunohistochemical methods of staining [20] are used for identification of collagen fibers, non-specific staining of the cell elements of the examined tissue sample is often possible as well as complication of the process of reaction setting-up requiring the application of two chromogens. Collagen is not a conservative protein: the diversity of its structural variants in different species of animals is thought to be caused by the variability of amino acid sequence and the collagen type [21]. In this connection, the selection of primary antibodies for each species and examined collagen type is labour-consuming and expensive.

On the contrary, the classical histological methods of staining intercellular substance of the connective tissue relative to the immunohistochemical ones have their advantages and may appear to be more suitable for the researcher. Owing to the versatility in the detection of various types of collagens and high affinity, the aniline blue in combination with pretreatment with phosphomolybdic or phosphotungstic acid is often used for collagen investigations in different organs and tissues [3, 5, 22, 23]. All these factors determined the choice of anilne blue a histochemical stain for the present method, which made it possible to detect specifically collagen fibers of the connective tissue. It has been established that our method using this stain is suitable for the combined application with immunohistochemical techniques for detecting macrophages and subsequent morphometric analysis.

## Conclusion

Optimization of the developed protocol for detecting Kupffer cells with antibodies to the Iba-1 microglial marker allows for simultaneous identification of the resident liver macrophages and collagen fibers without heat-induced antigen retrieval. The presented method of staining provides the possibility to perform effectively the morphometric analysis including binarization, color image segmentation, determining the area of the objects and their number, calculating the structure areas using a morphometric grid.
